# China's practical wisdom: Assumption of liability for endangering public health in bankruptcy proceedings—A case study of the Changchun Changsheng Biotechnology vaccine incident and the Johnson & Johnson baby powder incident

**DOI:** 10.3389/fpubh.2022.1003330

**Published:** 2022-11-09

**Authors:** Chaoyi Huang

**Affiliations:** School of Law, Chongqing University, Chongqing, China

**Keywords:** bankruptcy, public health, tort creditor priority, Changchun Changsheng Biotechnology, Johnson & Johnson, tortious liability

## Abstract

The assumption of liability for endangering public health has always been a legislative challenge in bankruptcy proceedings. Although it has been theoretically proven that the tort creditor should hold a position higher than that of unsecured creditors in bankruptcy proceedings, both legislation and judicial practice have been found wanting in many countries. China has witnessed large-scale domestic public health incidents where the tort debtor has entered bankruptcy proceedings while the tort claims were being settled. In Changchun Changsheng Biotechnology vaccine incident, to maintain social order and protect the rights and interests of the tort creditor, the Chinese government required the tort debtor to set up a special compensation fund of RMB 500 million and hand it over to a third party for management. This approach was mainly adopted because tort creditors can only participate in the bankruptcy distribution as an unsecured creditor, according to the Enterprise Bankruptcy Law of China, and as a result, their rights and interests cannot be guaranteed. In the context of the Enterprise Bankruptcy Law of China, this approach face predicaments of legitimacy and effectiveness. Moreover, even if the legislators follow scholars' advice and grant the tort creditor priority in bankruptcy proceedings, that would still not be enough to protect the rights and interests of the tort creditor, not to mention the possibility that the tort debtor might follow the example of Johnson & Johnson to avoid liability in practice. In fact, the Chinese government's approach is similar to that of Johnson & Johnson's, but more advisable. The Enterprise Bankruptcy Law of China (Bill of Amendment) will be submitted to the Standing Committee of the National People's Congress for preliminary deliberation this year, and the Chinese government's approach to the Changchun Changsheng vaccine case is very likely to be codified. This will resolve the predicaments of legitimacy and effectiveness that the government's current approach is facing and serve as a point of reference for the future revision of U.S. bankruptcy law and the handling of related cases.

## Introduction

Between 2014 and 2018, Changchun Changsheng Biotechnology (hereinafter referred to as “Changsheng”) blended two or more batches of a stock solution to prepare a freeze-dried rabies vaccine for human use (Vero cell) and created the batch numbers for the blended stock solution. From 2016–2018, Changsheng altered the batch numbers or actual production dates of 184 batches of the products involved, indirectly postponing their expiration dates. From March to April 2018, Changsheng produced nine batches of products using the expired stock solution created in 2017. In addition, Changsheng sent some poor-quality stock solutions with low antigen content for secondary concentration so that they would reach the preparation standards and then be used for production. To cover up these illegal actions, Changsheng destroyed the original production records and created false records. According to the National Medical Products Administration, Changsheng destroyed relevant evidence by replacing and processing the surveillance video memory cards and some computer hard disks (1).

### Timeline of events

- In July 2018, President Xi directed relevant government departments to handle sternly Changsheng's vaccine case to safeguard the legitimate rights and interests of the people (2).
- On July 24, 2018, all 15 people involved, including Gao Junfang, president of Changsheng, were detained by the Public Security Bureau of Changchun New District according to the law as suspects of criminal offenses (3).
- On August 16, 2018, Chinese Premier Li Keqiang held an executive meeting of the State Council in which the results of the investigation into Changsheng's problematic vaccine were shared and the relevant decisions were made (4).
- On October 16, 2018, Changsheng was given administrative penalties; its drug production license was revoked and it was fined 9.1 billion yuan by the Drug Supervision and Administration Department (5, 6). Consequently, on January 14, 2019, the Shenzhen Stock Exchange decided to delist the company's stock for serious violations. The company's stock entered the delisting clearing period on October 16, 2019 and was eventually delisted on November 27, 2019 (7).
- On October 16, 2018, Changsheng and China Life Insurance Co., Ltd. entered into the Entrusted Management Agreement on Compensation for Changchun Changsheng's Faulty Rabies Vaccine according to the Implementation Plan on Compensation for Changchun Changsheng's Faulty Rabies Vaccine that was jointly developed by the National Medical Products Administration, the National Health Commission, the China Banking and Insurance Regulatory Commission, and the provincial government of Jilin on October 12, 2018 (8). On October 22, 2018, Changsheng made a payment of RMB 500 million to China Life Insurance Co., Ltd. to set up a special compensation fund (9).
- On June 27, 2019, Changchun Intermediate People's Court accepted and heard Changsheng's bankruptcy liquidation case (10).

Notably, the fine (RMB 9.1 billion) imposed on Changsheng remains the highest imposed on pharmaceutical companies for illegal acts in China (11).

### Structure of this paper

The remainder of this paper consists of a chapter (Chapter 2) presenting reflections on the predicament of legitimacy and effectiveness facing approach adopted by Chinese government then three chapters (3, 4, and 5) on assessment of policy/guidelines options and implications, followed by Actionable Recommendations (Chapter 6), and the Discussion (Chapter 7). Chapter 3 analyzes the logic of the Chinese government's approach of handling public health liability in the Changsheng case. Chapter 4 compares the Changsheng case and the Johnson & Johnson case and concludes that they are essentially the same in dealing with public health liability, but that the approach of the Chinese government is likely to be more desirable. The fifth chapter analyzes why the Chinese government's approach is more worthy of recommendation.

Although the paper argues that the Chinese government's approach to handling the public health liability in bankruptcy proceedings is more commendable, as indicated in Chapters 2 and 3, there are also urgent problems to be solved in the Chinese Government's approach, which should be done in the aspect of legislation. This reflected in the sixth chapter (“Actionable Recommendations”). Finally, the “Discussion” chapter examines different reasons for the same choices in China and the United States. The paper contends that although the approach adopted in dealing with the problem of endangering public health in the Changsheng case and the Johnson & Johnson case in the United States are essentially the same, the factors behind the consideration are different. The Chinese government's approach is mainly based on maintaining social stability, not on improving the efficiency of society; however, it is argued here that the judges in the Johnson & Johnson case acted improve the efficiency of the society as a whole.

## Reflections: Predicaments of legitimacy and effectiveness facing approach adopted by Chinese government

After the Changsheng vaccine incident, Chinese people mainly focused on how to compensate the victims and how to punish those responsible, and few scholars in the theoretical field paid attention to whether the Enterprise Bankruptcy Law of China could serve as a basis for solving the problem of victim compensation. Although Changsheng finally entered the bankruptcy procedure, the Chinese government did not solve the problem of victim compensation within the framework of the Enterprise Bankruptcy Law of China, instead issuing special policies to solve the problem. Through the study of those policies and the judgments made by the courts, it can be concluded that there are many predicaments requiring urgent resolution, including both the legitimacy predicament and the effectiveness predicament.

The predicaments of legitimacy and effectiveness facing the Chinese government's approach stem from the Enterprise Bankruptcy Law of China does not have any special provision for the settlement of tort liability in bankruptcy proceedings, let alone for the assumption of liability for endangering public health. Some scholars who have carried out systematic research on the settlement of tort liability in bankruptcy proceedings in China opine that a certain proportion of the amount of the creditor's rights secured with property should be regarded as unsecured creditors' rights, and the secured property corresponding to the quota of the creditor's rights should be used prior to paying off tort liability against secured creditor (12). Wang Xinxin, a well-known bankruptcy jurist in China, holds the same view. He also points out that the scope of prior repayment of creditors' rights should be clarified (13). However, in the face of an incident endangering public health in Changsheng vaccine incident, instead of adopting the solutions proposed by scholars, the Chinese government used other approach without a bankruptcy law basis. The Chinese government did not adopt the scholars' proposals mainly because allowing the priority use of a certain percentage of the amount of secured claims for paying off the debts for personal tort would have had a negative impact on the stability of commercial transactions and would infinitely increase the costs of commercial transactions. Given the impact of the Changsheng vaccine incident in China and the fact that the tort debtor, Changsheng, entered bankruptcy proceedings, it is of great significance that the legitimacy and effectiveness of the relevant practices of the Chinese government—based on the provisions of Chinese law, in this case—are examined. Theoretically, the first things to be questioned are as follows: (a) What is the nature of the special fund set up by the Entrusted Management Agreement on Compensation for Changchun Changsheng's Faulty Rabies Vaccine between Changsheng and China Life Insurance Co., Ltd.; and (b) whether the special fund would be considered the property of the debtor after Changsheng entered bankruptcy proceedings. If yes, was it still considered a “special fund for a special purpose,” and could it be distributed to other creditors? If the special fund was considered a “special fund for a special purpose” and not the property of the debtor, what was the legal basis thereof? If it was not the property of the debtor, could the tort creditor get compensation from Changsheng's other property under the circumstance that the special compensation was insufficient in amount?

## The special fund for compensation was not the property of the debtor in nature, and the bankruptcy administrator had no right or need to exercise its administrative authority

On the question of whether the special fund is the property of the debtor, the documents issued by the Chinese government do not give a clear answer, but we can get the answer from the judgments of Chinese courts. The judgment in the case *Weihai Municipal Center for Disease Control and Prevention v. Changchun Changsheng* suggests that the tort creditor can only claim rights against China Life Insurance Co., Ltd. and not Changsheng. In the (2020) Ji 01 Min Chu No. 83 judgment given by Changchun Intermediate People's Court of Jilin Province for the case of *Weihai Municipal Center for Disease Control and Prevention v. Changchun Changsheng*, the court found the following:

On October 16, 2018, Changsheng signed the Entrusted Management Agreement on Compensation for Changchun Changsheng's Faulty Rabies Vaccine with China Life Insurance Co., Ltd. On October 22, 2018, Changsheng transferred the vaccine compensation of 500 million yuan to the account of China Life Insurance Co., Ltd., as agreed in the Agreement. Therefore, Changsheng set up the special compensation fund according to the instructions and entrusted China Life Insurance for management thereof. Even if Weihai Municipal Center for Disease Control and Prevention had corresponding expenses for subsequent and supplementary vaccination, the expenses should be covered by China Life Insurance after a due audit. Weihai Municipal Center for Disease Control and Prevention should not claim such expenses from Changsheng.

Based on said judgment and the relevant provisions on the centralized jurisdiction of bankruptcy cases in the Enterprise Bankruptcy Law of China (according to article 21 of the Enterprise Bankruptcy Law of China, bankruptcy cases are under the centralized government, i.e., after a debtor enters bankruptcy proceedings, all cases related to the debtor can only be heard by the court that accepted the bankruptcy case), after Changsheng entered bankruptcy liquidation in 2019, the compensation amount of 500 million yuan was not included in the bankruptcy estate. Otherwise, the court would not have dismissed the plaintiff's claims and asked them to claim their rights against China Life Insurance.

According to article 2 of the Implementation Plan on Compensation for Changchun Changsheng's Faulty Rabies Vaccine, the special compensation should be used to pay for any subsequent and supplementary vaccination damages, civil lawsuit compensation, damage determination, consultation services, clinical observation, and so on, occasioned by the faulty rabies vaccine. Therefore, the tort creditors could no longer claim rights against the tort debtor but against China Life Insurance. Further referring to article 5 of the Implementation Plan:

According to the characteristics of rabies that its incubation period is usually 1–3 months and rarely longer than 1 year, the inoculated people that suffer the damage listed in article 3 within 1 year from the date of inoculation should apply for determination and be compensated according to this plan; those who suffer damage after 1 year from the date of inoculation may file a civil lawsuit for compensation before the people's court according to law.

In this case, the tort debtor is Changsheng, and according to the principle of privity of contract (i.e., the contract cannot be an obligation for third parties here), the Entrusted Management Agreement between Changsheng and China Life Insurance only bound the contracting parties. So how could the tort creditors claim rights against China Life Insurance for the damages they suffered?

The challenges that the Chinese government's approach encountered in handling the public health incident by setting up a special fund can be split into two groups: (a) the predicament of legitimacy; and (b) the predicament of effectiveness. The former is expressed as the conflict between the Chinese government's approach and the Enterprise Bankruptcy Law of China and other laws, while the latter concerns whether the approach adopted by the Chinese government could effectively protect the interests of the tort creditor and achieve the expected effect—according to the Enterprise Bankruptcy Law—after the Changsheng entered bankruptcy proceedings.

### The approach faced legitimacy and effectiveness predicaments given that the special fund did not fall within the debtor's property

According to article 2 of the Implementation Plan, with respect to the entrustment relationship between Changsheng and China Life Insurance for the compensation of 500 million yuan, China Life Insurance was only the trustee rather than the owner. In other words, Changsheng was still the owner of the 500 million yuan. According to article 17 of the Enterprise Bankruptcy Law of China, after a debtor enters bankruptcy, the debtor's property holders should deliver the property to the bankruptcy administrator. In addition, article 2 of Judicial Interpretations (II) of the Enterprise Bankruptcy Law of China provides that whatever is not recognized as the debtor's property in accordance with the law and administrative regulations shall not be identified as the debtor's property. (i.e., laws and administrative regulations here refer to laws enacted by the National People's Congress of China and regulations enacted by The State Council of China) The Implementation Plan jointly formulated by relevant government departments in China is obviously not a law or an administrative regulation; therefore, it cannot be held as the basis for determining that the 500 million yuan was not the debtor's property. Nevertheless, the Chinese court ruled the opposite.

Moreover, according to an overview of provisions of the Enterprise Bankruptcy Law of China, Chinese legislators have strictly followed the rules that “property-secured creditor rights are paid first” and “unsecured creditors' rights are paid equally.” According to the Enterprise Bankruptcy Law, compensation, including medical expenses and damages, can only be listed as unsecured claims equivalent to general contractual obligations behind general priority. Debtor is not allowed to make any individual settlements on specific creditors when debtor close to bankruptcy (12). The decision of the Chinese court clearly contradicted the basic principles of the Enterprise Bankruptcy Law.

In addition, according to article 54 of the Enterprise Bankruptcy Law, after the principal (i.e., Changsheng, in this case) enters bankruptcy proceedings, if the entrusted party (i.e., China Life Insurance, in this case) has no knowledge of the aforesaid facts and continues to deal with the entrusted business, the entrusted party shall file their claim as a unsecured creditor in bankruptcy law. According to a reverse interpretation of this provision, if the entrusted party knows the facts, they should cease handling the entrusted matters, and the bankruptcy administrator should instead handle the same in accordance with article 25 of the Enterprise Bankruptcy Law. However, article 2 of the Implementation Plan stipulated that the compensation fund would be managed by China Life Insurance and specified that the management work would be supervised by the China Banking and Insurance Regulatory Commission. But if the compensation fund of 500 million yuan was not the debtor's property, what would be the legal relationship between the bankruptcy administrator and China Life Insurance and the China Banking and Insurance Regulatory Commission? According to article 25 of the Enterprise Bankruptcy Law, the responsibility of the bankruptcy administrator includes representing debtors in litigation, arbitration, and any other legal proceedings. If the 500 million yuan was not the debtor's property, the bankruptcy administrator would have no right to exercise any rights, including management and supervision, over the fund or to assume responsibility in order to participate in litigation, arbitration, or any other legal proceedings on behalf of the debtor. However, after the debtor entered the bankruptcy proceedings, the law did not authorize subjects other than the bankruptcy administrator to participate in the litigation, arbitration, or any other legal procedures involving the debtor. If the bankruptcy administrator was required to handle the litigation, arbitration, or any other legal proceedings involving the 500 million yuan, would the administrator be entitled to receive the corresponding remuneration? If so, what is the legal basis?

In sum, according to the provisions of the Implementation Plan, the bankruptcy administrator does not have the right to dispose of the compensation fund or to exercise supervision over the use of the fund, but this conflicts with the basic principles of the Enterprise Bankruptcy Law.

### The effectiveness predicament facing solving the assumption of liability for endangering public health in bankruptcy proceedings by setting up special funds

Returning to the case itself, the special fund for compensation was set up to avoid sudden management difficulties or any excessively speculative behavior on the part of Changsheng that would go against the protection of the rights and interests of the tort creditor. This approach may serve as a point of reference for future instances in which the tort debtor does not enter bankruptcy proceedings. However, once the tort debtor enters bankruptcy proceedings, it may become another situation altogether.

According to the Chinese government's policy and the judgment of the Chinese court, even though Changsheng entered bankruptcy proceedings, the tort creditor could still claim compensation from China Life Insurance rather than the debtor. The court ruled that Changsheng enter bankruptcy liquidation in June 2019. According to the Enterprise Bankruptcy Law, the debtor cannot be transferred to bankruptcy reorganization proceedings after being ruled to enter bankruptcy liquidation proceedings since such a debtor will cease to exist after the bankruptcy liquidation proceedings. Therefore, if the compensation fund of 500 million yuan that was set up by the debtor was not enough to compensate all the tort creditors, the tort creditor would not be able to claim any more compensation from the debtor. Consequently, the question that needs to be answered is of whether there is a better approach to remedying such situation.

## China's logic: Closing loopholes in legislation with power

The existing Chinese Enterprise Bankruptcy Law was promulgated in 2006 and implemented in 2007. Over the next decade, China's economy witnessed rapid growth, resulting in a very limited application of the Enterprise Bankruptcy Law. In this period, there were only a few hundred bankruptcy cases in China each year, to which academics paid little attention. In recent years, along with the supply-side structural reform (i.e., improve the quality of supply, advance structural adjustment through reform, correct distortions in the allocation of factors of production, and expand effective supply. It means inefficient firms will go into bankruptcy) proposed by the Chinese government and the spread of the COVID-19 epidemic, the number of bankruptcy cases has surged to tens of thousands (in 2015, 3,568 bankruptcy cases were accepted and heard in China; the number increased to 4,076 in 2016, 7,306 in 2019, and 13,369 in 2020). In this new context, legislators and scholars have begun to attach more importance to the Enterprise Bankruptcy Law (14). However, the lack of attention paid to the Enterprise Bankruptcy Law by legislators and scholars in China in the past has led to insufficient legislative efforts and limited theoretical research on bankruptcy law in China. For example, the Enterprise Bankruptcy Law does not offer a clear definition of the liquidation status of the tort creditor in bankruptcy proceedings, not to mention the protection of the rights and interests of the tort creditor after the tort debtor enters bankruptcy proceedings in cases of large-scale public health incidents. This means that the tort creditor in bankruptcy proceedings in public health incidents in China has the same status as the tort creditor in U.S. bankruptcy proceedings—they only receive partial compensation as unsecured creditors and have no room for bargaining (15). Some scholars have proposed that the claims to claim compensation for infringement of personal rights should be granted the same order of liquidation as labor claims in bankruptcy claims by offering judicial interpretations (16). However, legislators have not adopted this view. Therefore, it is unrealistic for the Chinese government to give the tort creditor priority in compensation after entering bankruptcy proceedings, and this may not necessarily achieve the objectives of the Chinese government. Considering all these factors, requiring (by administrative means) the debtor to set up a special fund to compensate the tort creditor seems to be the only viable solution.

We must admit that the approach of the Chinese government in the Changsheng case was, in its nature, a strategy adopted by the Chinese government and the judicial body to close the legislative loopholes in Enterprise Bankruptcy Law. What this strategy can be successful? This was possible because, according to the Enterprise Bankruptcy Law, the acceptance of a bankruptcy case and the handling of related derivative actions should fall under the jurisdiction of the same court. Therefore, once the Chinese government and the bankruptcy court reach a consensus, even if the tort creditor lodged a derivative lawsuit, the judge handling the relevant derivative actions would not make a judgment inconsistent with the Chinese government and the bankruptcy judge. In the meantime, considering the Chinese public's general recognition of priority compensation for infringement of the right to life and health, the public would not raise any objections to the handling of relevant bankruptcy cases (for instance, in the Sanlu milk powder incident, the Chinese government also skipped the provisions of the Enterprise Bankruptcy Law to guarantee compensation for the tort creditor) (12).

In addition, asking the debtor to set up a special fund to compensate the tort creditor helps maintain social order and appease the tort creditor. In the real world, people facing similar social instances in China often exert pressure on the government through non-legal means or even hold the government accountable, although they know that the tort debtor, not the government, is liable. The Chinese people act this way mainly because since ancient times they have believed that the government has an obligation to protect the rights and interests of its people, even if it is not the perpetrator of the illegal acts.

Finally, the requirement directing the debtor to set up a special fund enhances the convenience of the tort creditor and minimizes the social cost. Those affected by the Changsheng vaccine were found in many provinces and cities in China. Tens of thousands of people were inoculated with the faulty vaccine. If each tort creditor claimed rights against the tort debtor on their own, the tort debtor would be exhausted in fund and time, and the social cost would be enormous. The approach adopted by the Chinese government not only freed the debtor from the burden of litigation but also reduced the litigation cost for the tort creditor.

Furthermore, a special fund set up by the debtor—as ordered by the government, with the amount of the special fund determined by the government—is also conducive to solve the problem of protecting the infringed in the future. Such issue have always been challenging for legislators and jurists; whether in China or the United States, the theoretical field has not yet reached a consensus on how to solve it. Tort debtors are inherently profit seeking, and as a result, they will inevitably minimize the compensation provided to the tort creditors. Here, tort creditors include possible future tort creditors. Nevertheless, the government will naturally strive to protect the rights and interests of the tort creditors to preserve social stability and protect the interests of the people; here again, tort creditors include possible future tort creditors. Therefore, the government should be the one to decide whether to set up a special fund and also set the size of the fund, thereby protecting the interests of possible future tort creditors as much as possible. In conclusion, I do not think there is a solution that can completely solve the problem of protecting the infringed in the future, and the Chinese government's approach can solve the problem to the greatest extent.

## Reference value of China's approach to handling public health hazards: *Changsheng v. Johnson & Johnson*

As explained by Stacy L. Rahl, the United States has not specified enough rules to control the unethical behavior of companies, and social welfare could hardly compensate the tort creditors in full in mass tort cases; therefore, to resolve complex conflicts of interest and protect the rights of tort creditors, it would be more appropriate to give the courts considerable discretion. (17) In the United States, the assumption of liability for endangering public health in bankruptcy proceedings may conflict with not only the pursuit of the bankruptcy law for debtor relief, but also the protection of other creditors' rights and interests; moreover, it lacks a relevant legislative basis for government to intervene in such cases, for which the courts have most of the decision-making power over the handling of relevant cases.

Still, China's Changsheng vaccine incident is quite like the Johnson & Johnson baby powder incident in the United States, the latter of which began in 2017, when a Los Angeles, California, court ordered the company to pay $417 million in damages to a 63-year-old woman who developed ovarian cancer after years of using their talc-containing baby powder on her genital area. Talc is known to be susceptible to asbestos contamination, and asbestos, a natural mineral fiber, is considered a Class 1 carcinogen by the World Health Organization, so talc containing asbestos impurities can cause cancer. Johnson & Johnson has been facing allegations that its products cause cancer since 2016. On October 18, 2019, the Food and Drug Administration (FDA) found small amounts of asbestos in Johnson & Johnson's talc products, forcing the company to recall a batch of more than 33,000 bottles from the market. Johnson & Johnson paid more than $34.4 billion in compensation to consumers from 2016 to 2019 alone. In May 2020, the company said it would stop selling talc-containing baby powder in the U.S. and Canada. As of April 2021, there were nearly 30,000 pending lawsuits against Johnson & Johnson and its subsidiaries in U.S. courts. There is still controversy over whether the company's talcum powder contains asbestos, but there have been numerous lawsuits and huge payouts. On October 14, 2021, LTL, a new company spun off from Johnson & Johnson, filed for bankruptcy reorganization proceedings in the Federal Bankruptcy Court for the Western District of North Carolina. According to bankruptcy court records, Johnson & Johnson faced more than 38,000 cancer-causing lawsuits and $3.5 billion in awards and settlements before it filed for reorganization (15, 18).

Under the burden of lawsuits, Johnson & Johnson first created a new legal entity and transferred its infringement liability to it. In the meantime, a relatively small portion of the original company's assets was also transferred so that the company was divided into a new company with assets and a new company with liabilities, LTL Management (“LTL” in short). Later, the newly established subsidiary with liabilities filed for bankruptcy, thus effectively protecting Johnson & Johnson from the impact of the infringement liability. (16) The maneuver used by Johnson & Johnson was called the “Texas Two-Step.” This case is like the Changsheng vaccine case in that the tort debtor divested some independent assets from the original company to compensate the tort creditor, and the tort creditor could only claim rights against the divested assets. This way, the burden of lawsuits on the tort debtor was reduced and the overall social benefits improved. Moreover, in both the Johnson & Johnson case and the Changsheng case, the part separated from the debtor's property to compensate the tort creditors was working fund or unsecured property from the debtor; this is different from the viewpoint held by some scholars, which is that China should secure more protection for the tort creditors by reducing the interests of the secured creditor (12, 13). In both cases, the interests of the secured creditor in the bankruptcy proceedings were not compromised by the special protection for the tort creditors (18). The two cases are different in that the independent assets divested by Johnson & Johnson were injected into an all-new company, which then entered bankruptcy proceedings, whereas in the case of Changsheng, a special fund independent of the tort debtor was set up instead. According to Chinese law, the special fund could neither become an independent legal entity nor go through bankruptcy liquidation (19).

Johnson & Johnson's approach is similar to that of the Chinese government in terms of the intended purpose or social effect, which is why Judge Michael Kaplan of the Federal Bankruptcy Court for New Jersey approved LTL's bankruptcy reorganization plan on February 25, 2022. However, Johnson & Johnson's approach was more costly than the Chinese government's approach because LTL had to pay some fees, including the bankruptcy administrator fees, after entering bankruptcy proceedings, which in turn reduced the property used to compensate the tort creditors (The high cost of bankruptcy has long been criticized by not only American scholars but also Chinese scholars). The approach of the Chinese government, on the other hand, avoided fees such as high remuneration for the bankruptcy administrator.

How the Chinese government dealt with the Changsheng vaccine incident was also better than how Johnson & Johnson dealt with the baby powder incident since LTL's assets may were insufficient to compensate all the tort creditors. That is why it can be argued that Johnson & Johnson evaded some of its liability and deprived the rights of the tort creditors by what is known as the “Texas Two-Step” (20). To prevent latecomers from following the example of Johnson & Johnson, the House Judiciary Committee chaired by Representative Jerrold Nadler (D-NY) conducted a Mark-up Session and passed H.R. 4777, the Non-debtor Release Prohibition Act of 2021, out of committee for consideration by the full House of Representatives. H.R. 4777 would prohibit or severely limit the maneuver recently used by Johnson & Johnson (21). However, this problem is not associated with the approach adopted by the Chinese government because Johnson & Johnson, as a profit-making entity, certainly wanted to minimize the compensation it paid out, while on the other hand, Chinese government wanted to make sure that all the tort creditors were compensated so that the cost and pressure to maintain social order could be reduced. The tort debtor has an incentive to use bankruptcy proceedings to achieve what non-bankruptcy proceedings could not, thereby harming the interests of the tort creditors (22).

## Legal basis for solving the problem of attribution of liability for endangering public health in bankruptcy proceedings by setting up a special compensation fund according to a government decision

In the United States, many scholars believe that bankruptcy reorganization is an effective solution to mass torts; on top of this, they have proposed legislative optimizations (23). However, the current literature offers justifications from the perspective of the debtor (i.e., by focusing on how to better solve the problem of liability for the tort debtor rather than on how to better address the problem of public health accountability). Of course, if the tort debtor has going-concern value, legislators and judges should try to maintain its social value as much as possible; however, regardless of whether the tort debtor has going-concern value, the protection for the debtor should never override the liability for public health. Unfortunately, in both China and the United States, scholars in related fields have not attached due importance to the maintenance of public health. From the author's point of view, when an incident endangers public health, legislation should prioritize the solution to the problem of liability for endangering public health over relief for the debtor based on bankruptcy theory and legislation concerning debtor rescue. The government has always been the best candidate for maintaining public health; the debtor or the debtor's other creditors and shareholders cannot be the best candidates. During the process, it is still doubtful whether the court will prioritize the resolution of attribution of liability for endangering public health. This is because if the debtor chooses bankruptcy reorganization, the court should also consider the debtor's relief at the same time—after all, the debtor's relief is the core objective of the bankruptcy reorganization system.

Cases of bankruptcy involving the endangerment of public health also concern how to resolve the uncertainties in the assumption of liability for mass torts. Roe suggested that reorganization proceedings against the debtor should be adopted in mass torts case. During the reorganization, claims can be pooled and centrally administered in a manner analogous to the central administration by trustees of bond indentures or pension funds. Compensation methods similar to the variable annuity would reduce disparity in compensation among future tort claimants (24). Meanwhile, Roe also put forth that part of the future profits of the debtor should be included in the special fund. However, this approach risks tort creditors not receiving full compensation in the end. The profitability of the debtor is often difficult to calculate. If the debtor faces continued losses in the end, the legislator will have to make more-complex and less-operable regulations. In the bankruptcy cases of Changsheng in China and Johnson & Johnson in the United States, the set special funds came from the existing assets of the debtors (not including part of their future profits). In this way, the difficulty of handling relevant cases and the risk of the tort creditor not receiving full compensation in the end will both be reduced.

Admittedly, in both Changsheng's and Johnson & Johnson's bankruptcy cases, the tort creditor took a settlement status higher than other claims' (except for secured creditors, because in both cases, the property for paying off the tort creditor did not include the secured property), at least in terms of the separated special funds. This is why the author believes that the approach of the Chinese government lacks a basis in the Enterprise Bankruptcy Law of China. Theoretically, the tort creditors should have priority, mainly because such creditors are “passive creditors” (12). The attribution of liability for endangering public health in bankruptcy proceedings involves not only important human rights, such as the right to life and health, but also social stability. Therefore, it should be under special protection in law. That is, based on the point of view that rights, including the right to life and health, should be given a higher status than other rights and interests, the approach taken by the Chinese government in the bankruptcy case of Changsheng and the court judgment in the bankruptcy case of Johnson & Johnson are reasonable; in the future, targeted improvements should be made to the legislation.

It should also be explained in theory whether the proposed thinking for problem-solving applies to other types of mass torts. Here, the author's answer is no. This paper studies the attribution of liability for endangering public health in bankruptcy proceedings; the views and legislative suggestions put forward herein aim at the same. In the author's opinion, the legislation should distinguish mass torts that endanger public health from other types of mass torts. Chen Xiahong, a renowned expert in bankruptcy law in China, proposes that mass torts should be divided into those in traditional domains and those in emerging domains, with the former mainly involving mass torts endangering public health (25). However, Chen does not describe in detail how to handle them differently, nor does he elaborate, based on concrete cases, why they should be handled differently. According to Chen, the practice of the Chinese government in the bankruptcy case of Changsheng was similar to setting up bailout funds or trust funds under the leadership of the government (25). However, the actual practice of the Chinese government differed significantly from Chen's description. The biggest difference lies in the fact that the fund was set up by the Chinese government before Changsheng entered bankruptcy proceedings, not afterward through negotiation with the court and all interested parties, including other creditors (As we all know, any disposition of the debtor's property after the debtor enters bankruptcy proceedings is subject to a vote of all creditors). As a result, the practice of the Chinese government in this case conflicted with the provisions of the Enterprise Bankruptcy Law of China. In addition, debtors who enter bankruptcy proceedings for mass torts in traditional domains (mostly mass torts endangering public health) should be treated differently from those who enter bankruptcy proceedings for mass torts in emerging domains (for example, in the bankruptcy case of Kangmei Pharmaceutical, the debtor faced high compensation for false statements) for the following reason. In mass torts in traditional domains, there are many future creditors, and the tort creditors are often diversified, involving many people who know little about the law. In mass torts in emerging domains, however, the tort creditors often have relatively rich knowledge reserves and high risk-resistance. Therefore, the latter type of tort creditors should assume a higher duty of care: such creditors should bear certain commercial risks for their investment behaviors and be treated differently from creditors in mass torts in traditional domains.

Even if debtors in bankruptcy proceedings are allowed to solve the problem of attribution of liability for endangering public health by setting up a trust, they may find the problem trickier later, because if at some point the claims against the trust exceed the amount of money that the corporation must contribute to it, numerous plaintiffs may go either uncompensated or grossly undercompensated. If the trust cannot be restructured to alleviate the shortage, the corporation may need to increase its contributions. Such an increase in contribution may require a modification of the reorganized corporation's Chapter 11 plan, a matter governed by the Bankruptcy Code (17). Furthermore, bankruptcy proceedings mostly adopt the settlement program of the majoritarian institution; that is, in bankruptcy proceedings, the tort debtor and most of the other creditors, including the non-tort creditors, have most of the right to decide the attribution of liability for endangering public health by setting up a trust. This is apparently disadvantageous for the tort creditor, because in public health incidents, the more compensation the tort creditor receives, the fewer the benefits enjoyed by the tort debtor and other rights holders.

When addressing the attribution of liability for endangering public health, both bankruptcy liquidation and bankruptcy reorganization proceedings should adopt the solution of setting up special funds, as decided by the government. Indeed, the repayment for creditors in reorganization proceedings is generally higher than that in liquidation proceedings. However, when it comes to liability for endangering public health, the question is no longer of which can enhance the repayment rate of the creditors but of how to ensure that the tort creditors can be repaid in full. We learn from these two cases that both the approach adopted by the Chinese government and that adopted by Johnson & Johnson were based on fully compensating the tort creditors, even though this may not have been realized in the end. This paper aims to determine how to better ensure that the tort creditors receive full compensation using the approach of setting up a special fund. Of course, there could be circumstances in which the special fund set up after a public health incident cannot guarantee that the tort creditors will all be paid off in full. Only in such cases will the government or other public welfare organizations be needed to compensate the tort creditors for the losses suffered. However, the approach proposed herein—that the government decides whether to set up a special fund and sets its size—minimizes the possibility that the special fund will be unable to guarantee full compensation for all tort creditors. Naturally, whether the debtor goes into bankruptcy reorganization or bankruptcy liquidation, the remaining special fund (if any) may be distributed to other creditors according to the provision on the additional distribution of article 123 in the Enterprise Bankruptcy Law of China.

## Actionable recommendations: Codification of the approach for handling cases of incidents endangering public health in China

Some Chinese scholars believe that the claims of personal infringement should be given liquidation status second only to bankruptcy costs and public debt (According to the Enterprise Bankruptcy Law of China, bankruptcy costs and public debt have the first order of discharge), and they argue that if the establishment of the secured claims occurs after the claims of personal infringement, the latter should have priority over the former (26). This view is bound to increase transaction costs and count against the development of business innovation. Although there are many theoretical discussions on giving priority to the tort creditor in cases of incidents endangering public health, this priority is not actually given in either China or the United States because it might discourage the entrepreneurial spirit or prejudice the status of other priority holders, including the secured parties in bankruptcy proceedings, thus increasing the economic costs. Moreover, if the Enterprise Bankruptcy Law of China adopts the above scheme, relevant legislation in China would find it hard to prevent a Chinese version of Purdue Pharma's bankruptcy, thus damaging the procedural choice of the tort creditor (27).

In commercial bankruptcy practice, many creditors do not have the ability to negotiate with the debtor (28). The best way to solve incidents endangering public health is to take part of the property of the tort debtor to compensate the tort creditor. Legislators should also realize that if the right to divest compensation funds from the property of the tort debtor is given to the tort creditor, this is likely to damage the rights and interests of the tort creditor. The tort debtor, as a profit-seeking entity, would naturally seek to protect its own interests, and this can only be solved by giving right to a public body like government. What needs to be addressed in legislation is how the relevant practices can be codified and harmonized with the Enterprise Bankruptcy Law of China and other laws.

To solve the predicaments of legislation and effectiveness facing the Chinese government when dealing with incidents endangering public health, the Chinese legislature should specify that in the face of such incidents, relevant government departments will have the right to require the tort debtor to set up a special compensation fund based on the severity of the incident, and the management and supervision of such a fund would then be handed over to a third-party organization. In the meantime, relevant legislation should be clear about the criteria for determining the amount of the special compensation fund in incidents endangering public health in anticipation of a situation in which the special fund is insufficient to compensate the tort creditor. To further protect the rights and interests of the tort creditor, the Enterprise Bankruptcy Law of China should also clearly state that the special funds set up for compensation in incidents endangering public health are not the debtor's property; otherwise, the provisions made by the legislators on setting up special compensation funds will be meaningless. In addition, the Enterprise Bankruptcy Law of China should allow the tort creditor to file a lawsuit against the tort debtor after the tort debtor enters bankruptcy proceedings and ensure that the bankruptcy administrator remains the representative of the tort creditor. At the same time bankruptcy administrator should be entitled to receive remuneration from the special fund rather than the debtor's property; otherwise, it will be unfair to the debtor's other creditors.

## Discussion: Different reasons for same choices in China and the United States

It should be noted that the purpose of this paper was not to compare the relevant legislation in China and the United States, but to compare the bankruptcy cases of Changsheng to that of Johnson & Johnson. The United States is a country of case law, where court judgments, especially the reasoning therein, have an important influence on relevant future judgments. As such, in this paper the court judgment in the bankruptcy case of Johnson & Johnson was studied. China, on the other hand, is a country of statutory law. Moreover, this paper focused on the attribution of liability for endangering public health in bankruptcy proceedings in China, for which the discussion on related issues must be closely centered on the relevant legislation in China and policy documents promulgated by the Chinese government. More importantly, the reason that the bankruptcy case of Johnson & Johnson attracted broad attention is that the judge made a judgment at odds with public understanding and the core content of the judgment was not the interpretation and application of relevant provisions in the current U.S. law, but the reasoning of the judge.

In light of the political institutions and social environment of China, the Chinese government highly values social stability. As a result, the Chinese government becomes deeply involved in the handling of public health incidents to avoid shortfalls of funds for compensating tort creditors. By contrast, in terms of factors such as the political institution and social environment, the U.S. government will not, and has no sufficient legal basis to, become overly involved in cases to be decided by the court based on social stability and other considerations. Therefore, in cases in which bankruptcy proceedings are initiated for liability for endangering public health, the rights and interests of tort creditors in U.S. bankruptcy proceedings may be less protected than those in China. In bankruptcy cases, the court must consider not only the protection of the rights and interests of the tort creditors if the debtor chooses reorganization (in practice and as supported by many scholars in the United States, such cases should adopt reorganization proceedings instead of liquidation proceedings, because according to the bankruptcy law, the creditors' rights to repayment in reorganization will be greater than that in liquidation), but also debtor relief. Although the rights and interests of tort creditors in U.S. bankruptcy proceedings may receive insufficient protection when compared with Chinese bankruptcy proceedings, we must admit that the sound social security system in the United States can solve this problem to a large extent. In China, on the other hand, the emerging social security system means that the Chinese government must intervene early and strongly in bankruptcy cases involving liability for endangering public health. However, in practice, such intervention has no basis in bankruptcy law. Therefore, this paper recommends improving the relevant legislation in China in the future.

Many Chinese and U.S. bankruptcy law scholars call for tort creditor priority in bankruptcy proceedings, but neither Chinese nor U.S. legislators have adopted this philosophy. Although both Chinese and U.S. legislators hold the same opinion on this, it does not mean they make this decision for the same reason. Given the legal system and social condition of China, Chinese legislators' decision not to adopt tort creditor priority is based on the consideration of social stability (29). Even if tort creditor priority was adopted in China, for example, giving the tort creditor the same priority as the employee in bankruptcy proceedings (30), the tort creditor may also not get a full settlement, which may encourage the tort debtor to leverage the bankruptcy procedure to shrink from liability and force the tort creditor to impose pressure on the government through illegal means. The United States does not adopt tort creditor priority out of concern for the stability, but to maintain the stability and predictability of business activities, and few scholars consider social stability when suggesting tort creditor priority. Few U.S. bankruptcy law scholars consider social stability a factor mainly because people will not “seek the government for help in everything” in the United States, but in China, this is not the case (31).

The approach the Chinese government adopted in the Changsheng vaccine incident was mainly aimed at maintaining social stability while lowering the total social cost. As a result, the decisions of the Chinese government have not caused any social controversies because their primary goal is to protect the interests of individuals and maintain social stability, while reducing the total social cost is only a secondary goal and may not even be an expected outcome.

## Author contributions

The author confirms being the sole contributor of this work and has approved it for publication.

## Funding

This study was supported by Basic Research Funds for the Central Universities of China (Agency: Ministry of Education of the People's Republic of China; Grant Number: 2022CDSKXYFX009).

## Conflict of interest

The author declares that the research was conducted in the absence of any commercial or financial relationships that could be construed as a potential conflict of interest.

## Publisher's note

All claims expressed in this article are solely those of the authors and do not necessarily represent those of their affiliated organizations, or those of the publisher, the editors and the reviewers. Any product that may be evaluated in this article, or claim that may be made by its manufacturer, is not guaranteed or endorsed by the publisher.

## Author disclaimer

The views expressed herein are those of the author and do not necessarily reflect the views of the Chongqing University School of Law and the Ministry of Education of the People's Republic of China.
